# Understanding the Ontogeny of the Immune System to Promote Immune-Mediated Health for Life

**DOI:** 10.3389/fimmu.2015.00077

**Published:** 2015-02-23

**Authors:** Arnaud Marchant, Tobias R. Kollmann

**Affiliations:** ^1^Institute for Medical Immunology, Université Libre de Bruxelles, Charleroi, Belgium; ^2^Division of Infectious Disease, Department of Pediatrics, University of British Columbia, Vancouver, BC, Canada

**Keywords:** fetus, newborn, immune ontogeny, adaptive immunity, innate immunity, vaccines, microbiome, chronic disease

Understanding the ontogeny of the human immune system is a prerequisite to developing efficient and safe interventions aimed at providing long-term protection against infectious pathogens or inflammatory disorders originating early in life. The immune system of the fetus and the newborn infant had long been considered as “immature.” This insight is based on the observation that infectious diseases are often more severe or more prolonged in early life. However, studies performed in the 80s have shown that cells involved in innate immune responses develop as early as the fourth week of gestation in humans and that mature fetal T and B lymphocytes can be detected by the end of the first trimester of gestation already. More recent evidence indicates that fetal precursors are at the origin of adult tissue-resident macrophages. In addition, studies of immune responses to alloantigens and pathogens *in utero* demonstrated that effector and regulatory responses are operational during human fetal life, further indicating that the immune system is not fundamentally immature in early life, but simply differs from immune responses observed in later life. Studies are now needed to determine the signals involved in the regulation of the functional programing of immune cells in early life and to define how these signals could be targeted to protect against infectious diseases as well as chronic inflammatory disorders. The objective of the Research Topic is to provide an overview of the current state of knowledge of the ontogeny of the immune system and of the pathogenesis of important infectious and inflammatory diseases in early life. Four themes emerged from the excellent contributions that have been assembled here.

## Fetal Origin of Innate Cells

Recent studies indicate that a number of innate cell subsets derive from fetal progenitors and self-maintain throughout life. Vermijlen and Prinz reviewed the development of innate lymphocytes (1). The innate features of these lymphocytes include the recognition of molecular patterns, their rapid responses, and the fact that they do not undergo clonal expansion. Innate lymphocytes can express rearranged γδ or αβ (iNKT and MAIT cells) T cell receptors (TCR) or express no TCR (ILCs). The authors propose that some subsets are “more innate than others” because they develop in the fetal thymus and cannot be regenerated in the adult thymus. Whereas the signals required for the development of most subsets of innate lymphocytes remain unknown, cells expressing semi-invariant TCR develop under the influence of small metabolites associated with monomorphic presenting elements. The ontogeny of myeloid cells was reviewed by De Kleer et al. (2). Mouse studies have demonstrated that adult tissue-resident macrophages derive either from fetal yolk sac macrophages or from fetal liver-derived monocytes. In contrast, bone marrow precursors replenish dendritic cell subsets as well as inflammatory macrophages and myeloid-derived suppressor cells (MDSC) throughout life. Myeloid cells are differently programed in early life as compared to adult life and express different levels of effector molecules involved in tissue growth and remodeling, inflammation, and induction of adaptive immune responses. As reviewed by Debock and Flamand, a similar development influences the functional programing of T lymphocytes, where the balance between regulatory and effector responses changes between fetal life and adult life (3). Krow-Lucal and McCune discuss the fact that many of the functions of lymphoid and myeloid cells derived from fetal hematopoietic stem cells (HSC) are involved in barrier integrity and induction of tolerance and propose that immune maturation may proceed in a layered fashion with a fetal system predominating *in utero* and an adult system predominating later in life (4). In addition to differences in the origin of HSCs, several regulatory signals contribute to the maintenance of tolerance to non-inherited maternal antigens and to commensal bacteria in fetal and early post-natal life.

## Immune Regulation in Early Life

As observed at the maternal side of the materno-fetal interface, a number of potentially redundant regulatory mechanisms are operational in the fetus and the newborn infant that promote immune homeostasis *in utero* and in the early post-natal life. These mechanisms involve cellular components and soluble mediators. As reviewed by Gantt et al., among the cellular components, MDSCs are present at high frequencies in early human life and could therefore play an important role in immune homeostasis (5). Pettengill et al. reviewed current knowledge of soluble mediators regulating T cell responses in early life (6). The evidence indicate that several components promoting pro-inflammatory T cell responses, such as the complement system, are produced at lower levels in early life whereas regulatory mediators, such as adenosine, promoting regulatory T cell responses are produced at higher levels than in adult life. An immune environment programed toward regulation may have an important impact on the susceptibility of the fetus and the young infant to infectious pathogens and on their responses to vaccines. Although the evidence for this notion largely remains to be provided in humans, mouse studies of the ontogeny of intestinal innate immune responses indicate important changes in the expression of innate receptors and downstream signaling in epithelial cells. As reviewed by Hornef and Fulde, these changes probably play a critical role in the establishment of host–microbial homeostasis but may also play contribute to the susceptibility to enteric pathogens (7).

## Immunity to Pathogens in Early Life

Understanding the pathogenesis of infectious diseases in early life is required for the development of effective interventions protecting young children but also sheds light on the ontogeny of immune responses *in vivo* in humans. A number of intracellular pathogens cause more severe disease or replicate at higher levels in the fetus and young child as compared to the adult. This reduced pathogen control could be related to a defect in the development of cell-mediated immune responses. Muenchhoff et al. review the evidence suggesting that the increased viral replication and rapid disease progression associated with pediatric HIV infection may involve reduced Th1 responses and increased regulatory T cell responses (8). In addition, they suggest that the relatively low frequencies of memory CD4 T cells present during the first months of life may reduce the HIV reservoir and may thereby provide opportunities for curative interventions. An unbalanced T cell response may also play an important role in the increased susceptibility of young children to RSV infection. As reviewed by Lambert et al., dysregulated Th2 and Th17 responses may limit viral control and promote inflammatory responses in the airways of RSV-infected children (9). Huygens et al. reviewed the studies of congenital CMV infection indicating that high frequencies of antiviral γδ and αβ T cells can be induced early during fetal life (10). Recent studies further suggest that functional exhaustion may be an important factor regulating effector T cell responses in early life. On the other hand, Bertoletti and Hong propose that the different clinical manifestations of HBV infection in young infants and in adults are related to differences in the host ability to trigger inflammatory responses rather than to immunological tolerance to the virus (11). These reviews provide a framework to further delineate protection from disease due to intracellular pathogens in early life. If common principles directing immune responses in early life are emerging, the immunological determinants of the clinical expression of infections probably involve pathogen-specific factors. This notion is illustrated by the studies of GBS infection. As reviewed by Landwehr-Kenzel and Henneke, the innate immune mechanisms involved in the control of the GBS niche at the mucosal level may also play a central role in the immunopathology associated with invasive infections and subtle changes in this balance may be critical to the susceptibility of newborn infants to GBS sepsis (12).

## Genes and Environment

As discussed by Newport, most immune responses show wide-ranging inter-individual variation within a population, indicating a multifactorial etiology where both genes and environmental factors interact (13). Vaccine studies provided evidence that genetic factors are important determinants of immune responses in early life. However, our understanding of the genetic factors involved in the control of immune responses to vaccines and infectious pathogens in infants remains limited. At the same time, a large number of environmental factors can modulate immunity during fetal and early post-natal life. As reviewed by MacGillivray and Kollmann, these factors include nutrients, chemicals, commensals, and infectious pathogens (14). The consequences of the exposure to these environmental factors can be short-term or have lifelong implications. As the environmental factors profoundly evolve between fetal life and adult life, the immune system is likely to be programed to have the capacity to adapt to these changes. One striking example of an evolving environment is the establishment of the microbiome. Microbial colonization begins at birth and the content of the microbiome continues to evolve during the first years of life. These evolving microbial signals could play a central role in the evolution of the functional programing of immune cells during this period. As reviewed by Arrieta et al., imbalances of the gut microbiome in early life are associated with the development of several chronic inflammatory disorders, including inflammatory bowel diseases and asthma (15). Asthma is a complex disease with multiple factors acting in concert over long time periods. In their review, Walker et al. characterized the complexity of the asthma phenotype and propose that prospective birth cohorts recording large datasets of clinical, physiological, biochemical, immunological, and microbiological markers over time are required to gain new insights into the pathogenesis of asthma (16). Although the Research Topic does not include contributions on other chronic inflammatory disorders that may originate in early life, it is likely that similar approaches will be required to unravel their complex pathogenesis. In addition to the financial and logistical challenges of such studies, tools to support the complex data analysis and statistical modeling will have to be developed. Another critical factor forming the immune environment of the fetus and the young infant is composed by the maternal antibodies transferred through the placenta and the breastmilk. As reviewed by Niewisk, maternal antibodies not only protect against pathogens to which the mother has been exposed but also decrease responses to many vaccines through complex interactions with infant B cells (17). A better understanding of the transfer of immunity from the mother to the young child is likely to open new avenues for the prevention of infectious diseases in early life through maternal and neonatal immunization.

The rich series of manuscripts included in this Research Topic only covers a fraction of the emerging knowledge about immune ontogeny and its role in the promotion of health in early life. Yet, it clearly indicates that this field of research is developing more actively than ever and that fetal life and early infancy are increasingly recognized as a critical period to shape the immune system for life. This has similarly been recognized in the “1000 days” (i.e., from conception to age 2 years) campaign (18) and the WHO’s Every Newborn Action Plan (19). Together with the manuscripts in this Research Topic of Frontiers in Immunology, early life can be seen not only as a window of vulnerability to infectious morbidity and mortality but also as a window of opportunity to promote immune-mediated health for life. As emphasized by Christopher Wilson, translating emerging biological knowledge into new and effective interventions will require that immunologists leave their silos and build bridges across a wide range of disciplines (20). We thank all the authors of the manuscripts for their very generous contributions to this project.

## Conflict of Interest Statement

The authors declare that the research was conducted in the absence of any commercial or financial relationships that could be construed as a potential conflict of interest.
